# Human performance in three-hands tasks

**DOI:** 10.1038/s41598-021-88862-9

**Published:** 2021-05-04

**Authors:** A. Noccaro, J. Eden, G. Di Pino, D. Formica, E. Burdet

**Affiliations:** 1 NEXT: Neurophysiology and Neuroengineering of Human-Technology Interaction Research Unit, Università Campus Bio-Medico di Roma, Rome, Italy; 2 Department of Bioengineering, Imperial College of Science Technology and Medicine, London, UK

**Keywords:** Biomedical engineering, Learning and memory, Neuroscience, Cognitive control

## Abstract

The successful completion of complex tasks like hanging a picture or laparoscopic surgery requires coordinated motion of more than two limbs. User-controlled supernumerary robotic limbs (SL) have been proposed to bypass the need for coordination with a partner in such tasks. However, neither the capability to control multiple limbs alone relative to collaborative control with partners, nor how that capability varies across different tasks, is well understood. In this work, we present an investigation of tasks requiring three-hands where the foot was used as an additional source of motor commands. We considered: (1) how does simultaneous control of three hands compare to a cooperating dyad; (2) how this relative performance was altered by the existence of constraints emanating from real or virtual physical connections (*mechanical constraints*) or from cognitive limits (*cognitive constraints*). It was found that a cooperating dyad outperformed a single user in all scenarios in terms of task score, path efficiency and motion smoothness. However, while the participants were able to reach more targets with increasing mechanical constraints/decreasing number of simultaneous goals, the relative difference in performance between a dyad and a participant performing trimanual activities decreased, suggesting further potential for SLs in this class of scenario.

## Introduction

More than two limbs are often required to successfully carry out complex tasks such as hanging a picture, industrial assembly or laparoscopic surgery. These activities are typically completed through coordination with other individuals, however a companion is not always available and miscommunications and a lack of familiarity between collaborators may compromise the task’s success and result in inefficient performance^1^.

*Supernumerary robotic limbs* (SL), which are (e.g. wearable) user-controlled robotic limbs, represent one potential means of allowing a single person to execute complex actions by augmenting their natural motor capabilities^2,3^. Many supernumerary devices, including supernumerary fingers^4,5^, arms^6,7^ and legs^8^ have been built across industrial and medical applications. Significant research has focused on the development and control strategies for these devices^9–11^. However, less research has been placed on two central questions for their application: *Are humans able to reliably make use of three limbs in task augmentation scenarios?* and *Which tasks are best suited to augmented activities?*

Previous studies which have evaluated the ability for a single human subject to coordinate three hands together (*trimanual activities*) include^12^, who studied re-sizing^13,14^, who demonstrated the ability to control three hands with the natural limb and^15^ who investigated the impact of adding a third hand to different bi-manual tasks of varying coupling level. From these studies it has been observed that human subjects can control three hands simultaneously. When the hands are uncoupled and free of any mechanical constraint, the addition of a third hand into the task has little negative impact on the subject’s performance when compared to their performance in similar bi-manual operations. In contrast when mechanical constraints were present between the hands, the addition of a third handed task was found to significantly degrade the subject’s performance in the original mechanically constrained bimanual task. While these studies have performed preliminary analysis into the trimanual skills of human subjects, they have to date been limited to applications in which the task could be performed with two hands. More importantly, they have not compared the subject’s performance to that which would be possible with traditional human-human coordination.

In this work, we investigated the questions of: (1) *how do subjects perform in different trimanual tasks with different levels of virtual mechanical coupling and simultaneous targets to track?* and (2) *how does the subject’s performance compare to a dyad executing the same task*? Using the subject’s dominant foot as an input for controlling a virtual third hand, thirty participants performed trimanual tasks in three scenarios: (1) uncoupled case, where each limb aims at a different goal and moves independently from the other limbs; (2) 2-coupled case, where two limbs have the same goal and are virtually mechanically coupled together while the third limb’s motion and goal are independent; (3) 3-coupled case, where all limbs have the same common goal and are virtually mechanically coupled. The subject’s preference and performance carrying out the task on their own and with a randomly assigned partner were evaluated using their task score and other motion characteristics. To our knowledge, this study is the first to compare solo and dyadic performance in trimanual activities. The study also builds on the exploration of mechanical and cognitive constraints from^15^ by considering additional scenarios. Virtual springs connecting the virtual hands were used to render the virtual mechanical coupling, avoiding cursors to be passively driven by a rigid connection.

## Materials and methods

### Experimental setup

**Figure 1:**
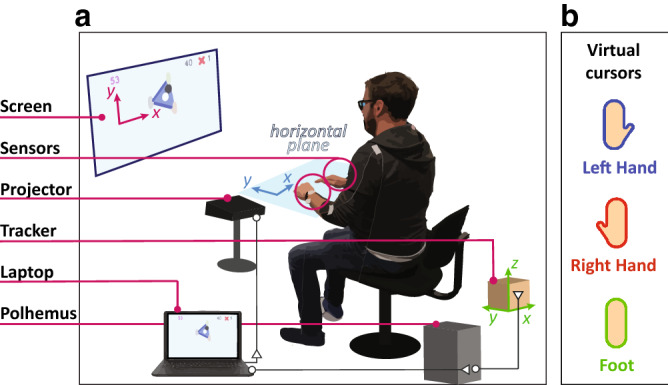
(**a**) Experimental setup: the hands and foot movements are tracked using the Polhemus Liberty tracking system. (**b**) Virtual cursors.

Figure 1 depicts the experimental setup. The participant/s were seated and their hand positions and foot position were acquired using three sensors from the electromagnetic Polhemus Liberty tracking system. The sensors were placed on the wrists and the dominant foot using a 3D printed holder with a layer of foam and velcro straps. To avoid foot fatigue, the foot was also fixed to a slippery shoe liner which they could use to slide against the floor. The tracker itself was placed behind the participants such that its y-axis pointed towards the screen and the z-axis pointed up. The participants faced a wall in which the 2D task feedback was projected.

The projected virtual cursor positions were displayed as smoothed rectangles with a colored contour and a thumb that recalls the shape of right and left hand (Fig. 1b). The positions of these cursors were controlled through the movements of the participant’s limbs, where movements in the horizontal plane were mapped onto the vertical plane projected display. Lateral movement corresponds to movement in the screen’s x-axis, whereas movement to/from the body is mapped into the screen’s y-axis. The movements of both the hands and the foot were not constrained, however, up/down motions did not produce any virtual cursor movement.

To optimally render the elastic coupling in the virtual environment, the final virtual cursor position was manipulated through the application of a virtual force derived from the sensor position measurements. This force was computed in a two stage process. First, to allow each subject to cover the whole virtual workspace, the real displacement of the hand (or foot) with respect to its initial position along the horizontal plane was mapped to the virtual environment through the hand and foot scaling factors, $s_h$ and $s_f$, respectively. The scaling factors were computed by estimating the limb range of motion for each participant as a function of their height *h*, such that

1 $$\begin{aligned} s_h = \frac{0.315}{8}\frac{h}{SD}\,, \quad s_f = \frac{0.246}{8}\frac{h}{SD}\,, \end{aligned}$$

where *SD* denotes the minimum screen dimension, $\frac{1}{8}$ was used to ensure the workspace lay within the desired screen operating range and the constants were determined according to average anatomical details^16^. Second, the resultant desired virtual position was mapped into virtual force through

2 $$\begin{aligned} F = k_{sg}\,(p_d-p)-k_{dg}\,v \,, \end{aligned}$$

where $p_d$ represents the desired cursor position, measured from the sensors and scaled, and *p* represents the actual cursors position; *v* is the current cursor velocity; $k_{sg}$ and $k_{dg}$ are the proportional and derivative gain values. To ensure numerical stability throughout operation, the gains $k_{sg}$ and $k_{dg}$ were computed using implicit Euler formulation as follows:

3 $$\begin{aligned} k_{sg}= & {} k_p \,g\,, \nonumber \\ k_{dg}= & {} (k_d+k_p\,dt)\,g\,, \nonumber \\ g= & {} 1/(1+k_d\,dt+k_p\,dt^2)\,, \end{aligned}$$

with $k_p=500$ N/m, $kd=1.5\,\sqrt{k_p}$ and $dt=0.02$ s the time between two following frames. The virtual environment was built in Unity. Both the scale factors and stiffness values were subjectively chosen based on a pilot test on three participants not included into the study. In particular, the force control gains were chosen to have a fast and stable cursor response, that matched the subject’s motion. Then the stiffness values of the elastic bands were adjusted depending on those gains values.

### Experimental protocol

Thirty participants, organized into fifteen dyads participated in the experiment. The experiment was approved by the Imperial College Research Ethics committee and carried out according to the Declaration of Helsinki. All participants gave their informed consent prior to starting the experiment.

**Figure 2:**
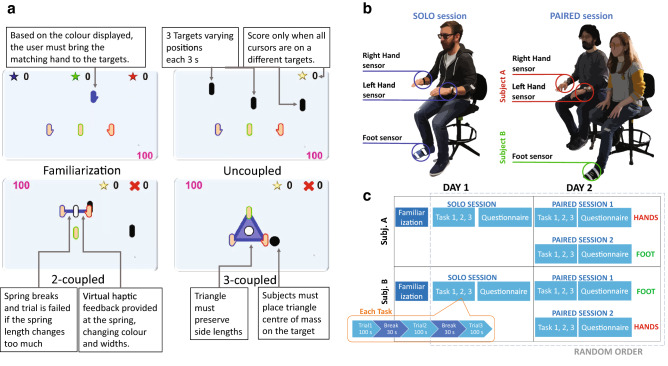
(**a**) Virtual tasks: Familiarization: one target appears at a time, with the same colour and shape of the tracking limb; Uncoupled Task: three black targets appear, each cursor has to reach a different target; 2-coupled Task: the two hands are virtually coupled by means of virtual spring. Two targets appear and have to be tracked with the foot and spring center of mass, while the spring length must be maintained. 3-coupled Task: One black target appear at a time. This must be tracked with the center of mass of the three coupled limbs without overstretching/over-compressing. (**b**) Participants performing the solo and paired sessions. (**c**) Experimental protocol: Each subject (Subj. A and Subj. B) performed three sessions across 2 days. Familiarization was always first, then the Solo and Paired session order was randomised. Each session considered each task, first the uncoupled and then the other two randomly arranged. Each task was repeated three times.

The participants (twelve women, $26 \pm 4.5$ years old) had to ‘catch’ the targets each with their hands and foot. Three tasks corresponding to different coordination scenarios were tested (Fig. 2a): (1) three uncoupled hands, with three different targets; (2) two hands virtually coupled plus uncoupled foot, with two different targets; and (3) three limbs virtually coupled with one target. The tasks were two dimensional, employing only two translational directions and no rotations. No physical springs connected the participants’ hands or their hands and the foot; instead, for tasks with coupling, the virtual cursors were coupled by means of a virtual elastic band.

The dyads’ partners (i.e. Subject A and Subject B) were selected randomly. Each dyad’s member performed three sessions in a random order (Fig. 2c):

- Solo Session: Subject A/B controlling the three cursors with the hands and the foot.
- Paired session 1: Subject A controlling the hands and B the foot.
- Paired session 2: Subject B controlling the hands and A the foot.

Each session considers the three tasks {uncoupled, 2-coupled, 3-coupled}. The task order was set so that the uncoupled task was first and the remaining tasks completed in random order. This was chosen to not bias participants with the coupled movements required in the other tasks before they executed the uncoupled task.

The three sessions were conducted in a random order across two days: on day 1 participants performed an initial *familiarization* before either their single *solo session* or two *paired sessions*. The remaining session/s were performed the next day. At each session’s end the participants filled a *questionnaire* rating their perceived difficulty and preference.

Each task was performed three times, each repetition (hereafter called *trial*) lasting 100 s followed by a 30 s break. In all tasks, participants were required to track a set of targets within a maximum time period of 3 s. If they succeeded in tracking a target, defined as being within a 1 cm of the target, they received a point and a “coin” sound was played, whereas if they broke a virtual spring a failure was recorded and a “buzzer” sound effect was played.

Throughout a trial, the participants were not allowed to communicate verbally. The virtual cursor was allowed to move throughout the whole rectangular screen, however, targets were limited to appear only in a centerd $30 \times 30\,\hbox {cm}^2$ square. In the dyad, each participant sat on either the right or the left side. To rule out the seating position from having an effect on the selection of targets on one side of the screen, participants kept the same chair during all sessions (Fig. 2b).

A detailed description of the familiarization phase and the experimental tasks is provided in the following subsections. A demo of the implemented tasks can be found at https://youtu.be/EYilJKLvtxA.

#### Familiarization

In this phase, there is no coupling between limbs and targets appear one at a time. The targets have the same colour and shape as the limb that has to reach it. The aim of the task is to reach as many targets as possible in 100 s with the corresponding limb. If a target is not reached within 3 s, it disappears, and a new target appears on the screen with a random colour corresponding to the tracking limb. The score is computed by summing the number of targets reached with each limb.

#### Uncoupled task

The hands and foot move independently from each other. Similarly to the familiarization, the aim is to reach as many targets as possible in 100 s. Three black targets are presented simultaneously and the participant can choose the cursor associated to each of the three targets. The score increases when each cursor is simultaneously located within 1 cm from a different target. After 3 s, the targets reset in different positions. Each set of targets represents a *sub-trial*, so that the score increases for each completed *sub-trial*.

#### 2-coupled task

This task includes a virtual spring connecting the two virtual hands corresponding to the participant’s hands, whose center is represented by a white cursor. Two black targets appear on the screen. The targets must be reached by the spring center and the virtual cursor associated with the foot. There is no cue nor constraint on which cursor has to reach which target, but to get the score point, the cursors need to be within 1 cm from the respective target at the same time.

In addition to tracking the targets, the participants also need to prevent the virtual elastic band from changing its initial length by more than 30%. This represents the mechanical constraint. Each band break due to stretch or compression is counted as a fail. The final score is given by the difference of completed sub-trials and the number of fails.

Visual feedback simulating haptic feedback is provided at the spring: the virtual band changes colour and width depending on the distance between the two connected virtual hands. Cursor distance $\Delta x$ is converted into elastic force $F_e=- K_e \,\Delta x$, acting on the cursors. The spring stiffness $K_e$ is set to 250 N/m for traction and 100 N/m for compression, to achieve a smooth rendering that recalls the natural feeling of an elastic object.

#### 3-coupled task

The three virtual limbs are all mechanically coupled to each other through a virtual triangular elastic band. Each limb holds one corner of the triangle. The aim is to put the center of mass of the triangle (highlighted with a white cursor) on the target. The triangle is defined by the area within the three springs that connect respectively the two virtual hands, the right hand and the foot, the left hand and the foot. The three springs must preserve, within a 30% range, their initial lengths to avoid task failure. Moreover, the hands must keep the center of mass within the elastic area. Virtual haptic feedback is provided through the triangle in the sense that the force in the spring for each length is shown by the colouring and the width of each spring.

## Data analysis

The participant’s behaviour is evaluated in terms of the task performance, motion characteristics and subjective experience questionnaire. The evaluation uses the following indices:

- *Score*: the number of completed sub-trials minus the number of fails (fails only apply in 2-coupled and 3-coupled tasks). We also evaluated separately the positive number of completed sub-trials, without considering fails.
- *Normalized sub-trial time*: average of the time required to complete a sub-trial divided by the initial distance between cursor and target.
- *Normalized travelled distance*: the total distance travelled by each virtual cursor on the screen, divided by the total number of sub-trials.
- *Smoothness*: the spectral arc length^17^ of the speed magnitude of each cursor.
- *Spring length variation*: the root mean square of the length variation of the connecting spring (in the 2-coupled task) or the root mean square of the length variation of the three connecting springs (in the 3-coupled task).

*Score* and *Normalized sub-trial time* measure the global task performance, whereas *Normalized travelled distance* and *Smoothness* reveal the motion characteristics of single limbs. *Spring length variation* finally is an index of *coordination*: the more two coupled limbs are coordinated in their movements, the more they preserve the initial spring length.

From the questionnaire, the preference and difficulty rank were computed both for the three tasks and for the three sessions (solo, hands control, foot control).

A 2-way RM ANOVA was used to analyze the measures of the *Score* and the *Normalized sub-trial time* needed to reach the targets; the factor analyzed were the Task {uncoupled, 2-coupled, 3-coupled} and the Control {Solo, Couple}. To analyze the Smoothness the Limb Combination was added as a third factor (with 3 levels: {Dominant Hand-Non Dominant Hand (DH-NDH), Dominant Hand-Dominant Foot (DH-DF), Non Dominant Hand-Dominant Foot (NDH-DF)}). Mauchly’s test was used to check data sphericity, eventually corrected using the Greenhouse–Geisser correction. Score, Spring Length Variation, Smoothness and Normalized Traveled Distance were analyzed with a Durbin test, after identifying their non-normal distributions by means of Shapiro–Wilks test. Post-Hoc tests were corrected with Bonferroni correction. In case of non-normal data post-hoc analysis was performed using Wilcoxon test. These analyses were conducted on the last trials only, in order to rule-out the learning effect which was assessed by means of a paired t-test comparing the first and last trial of each task. For the same reason the familiarization was not considered into the statistical analysis. Questionnaire ranks were compared using Friedman and Wilcoxon tests. Only the statistically significant *p*-values are reported and all refer to a significance level of 0.05.

## Results

### Task performance

**Figure 3:**
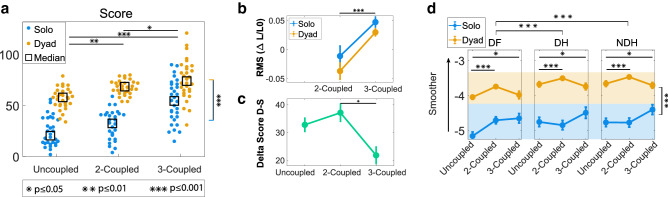
Performance: (**a**) Score for Uncoupled, 2-Coupled and 3-Coupled task. Each dot represents the value of the last trial. Blue dots correspond to solo sessions; orange dots correspond to paired sessions; squares represent median values. (**b**) Spring length variations ($\Delta L/L_0$) in 2-Coupled and 3-Coupled tasks. (**c**) Delta score between Dyad and Solo. (**d**) Limb’s Smoothness (spectral arc length) for Uncoupled, 2-Coupled and 3-Coupled task (DF: Dominant Foot, DH: Dominant Hand and NDH: Non-Domimant Hand). Each dot represents the average value of the last trial, while bars represent the standard error. The ‘*’ symbol is used to mark the statistically significant results, with * for ${p}\leqslant 0.05$, ** for ${p}\leqslant 0.01$ and *** for ${p}\leqslant 0.001$. All reported *p*-values correspond to a significance level of 0.05.

Figure 3 shows the subject performance for each of the three tasks in terms of their score and (where applicable) spring length variation and smoothness. It can be seen that dyads perform better than solo subjects in term of score (${p}<0.001$) and that their performance is significantly better in the 3-Coupled task when compared to both the uncoupled task (${p}<0.001$) and 2-Coupled task (${p}=0.03$) (Fig. 3a). These results are confirmed looking at the number of completed sub-trials, not considering the fails: in all tasks the solo subjects completed less sub-trials than dyads (${p}<0.001$) and the 3-Coupled task shows significantly more sub-trials completed than the 2-Coupled task, which in turn has more completed sub-trials than the uncoupled task (all *p* values lower than 1/1000). This suggests that in general people controlling three limbs track the targets more efficiently in the 3-Coupled task than in the others.

Conversely, the subjects seemed to struggle with the virtual mechanical constraints, as emerges from the length variation of the elastic band (Fig.  3b), that is higher in the 3-Coupled task than in the 2-Coupled task. In coupled trials, the subject controlling the hands tended to make them close to each, compressing the elastic band (negative values); on the contrary, in the 3-Coupled task the band is almost always overstretched in both the solo and dyad conditions (positive values). Considering the absolute variation rate, there is no difference between solo and dyad conditions nor between the tasks. The comparison between the first and last trials, in term of score and normalized sub-trial time, revealed a learning effect in all the tasks (${p}<0.01$).

**Figure 4:**
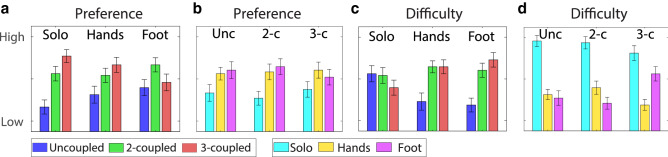
Questionnaire results: (**a**,**c**) preference and difficulty rank among the tasks for the solo and paired (hands-foot) sessions; (**b**,**d**) Preference and difficulty rank among the sessions for the three tasks.

### Motion characteristics

The smoothness across different sessions can be seen from Fig. 3d. The solo sessions are less smooth than the dyad ones (${p}<0.001$) and they are significantly smoother in the 2-Coupled and 3-Coupled tasks than in the uncoupled task. Across all trials the foot exhibits significantly lower smoothness (${p}<0.001$), however, no significant difference was observed in the virtual motion resulting from the two hands.

Similar results were also observed in the distance travelled by each virtual cursor. In particular, dyads again perform better than solo subjects: the distance travelled by the cursors on the screen (divided by the number of total sub-trials) is in all cases lower for the paired sessions than for the solo one (${p}<0.001$). Additionally, the distance travelled by the foot controlled cursor is significantly different (higher) than that of the hands (${p}<0.001$). However, in this case the dominant hand covers significantly less distance than the other limbs (${p}<0.001$).

### Questionnaire assessment

The questionnaire reveals that most of the subjects prefer the 2-Coupled and 3-Coupled task rather than the Uncoupled task (${p}<0.01$) even though they find them more difficult than the Uncoupled task (${p}=0.015$, ${p}=0.029$). In general, the uncoupled task is the least preferred in any case—solo, hands and foot session (Fig. 4). Subjects also preferred to work in dyads rather than alone -preference for the solo session is significantly lower than preference for the hands session (${p}=0.011$) and for the foot session (${p}=0.033$)- perceiving the solo session significantly more difficult than the paired ones (${p}\ll 0.001$). There is no clear preference for the foot control rather than the hands (Fig. 4).

## Discussion

### Task performance

Throughout the experiment, participants performed better in dyads than in solo, in terms of their score, travelled distance and smoothness, indicating that they possessed more control in trimanual activities for the different tasks. In general, this suggests that the benefits of having the task performed by two agents, such as parallelised planning, outperformed any of the negative effects coming from the need for communication between the two partners. However, it should be noted that in most tasks, where hands and foot controlled virtual limbs were not coupled, the dyads could complete the task independently of their partner. Since the participants possess everyday experience in bimanual/unimanual actions, they may be starting at a higher skill level than the solo scenario. Therefore further study of the learning effects is required.

Another aspect to be eventually investigated is the coupling between one hand only and the foot, with the other hand uncoupled. That case was not considered here for two main reasons: (1) to limit the number of different tasks (thereby ensuring the sessions were within a 90 minutes period); (2) because the current choice seemed to be most realistic case for applications (where the user completes a task using their hands while they can do an additional task with an extra limb).

Compared to the hands, the higher distance travelled by the foot and its lower smoothness are coherent to its use requiring lower fine motor control during everyday tasks. While the use of non-equal scaling factors allowed for equivalent workspace sizes for the different limbs, it is also possible that the scaling factor acted to further enlarge this difference. This suggests a limit for foot control as a tool for trimanual tasks, requiring either significant training in the use of the foot to normalise the virtual hand dexterity, software adjustment, the use of an entirely different input device for the third hand or the integration of human-robot collaboration strategies to optimise the foot control’s performance^18,19^.

The participants’ score increased in the 3-coupled task when compared to the other tasks. This may be a result of required lower cognitive load, where this task requires subjects to only focus on one goal at a time. Also, since the foot exhibits lower dexterity, this coupling with the hands could have contributed to improve the participants’ performance in the 3-coupled task. However, it should be noted that the coupled tasks possess the additional factor of the virtual spring constraint. While the absolute spring variation did not show a significant difference depending on the addition of more springs (as between the 2-coupled and 3-coupled tasks), its addition represents a critical factor of successful task performance that cannot be directly considered within the score.

While the dyad performance across the different tasks is observed to always be better than that of the solo subject, the difference in performance between the solo and dyad sessions was found to be smaller for the 3-Coupled task (Fig. 3c) when compared to the two-Coupled task ($p=0.024$). This result suggests that the 3-coupled case may be most suitable trimanual task for a single operator since it requires continuous coordinated motion across the limbs. This effect may derive from the virtual mechanical constraints that could have helped the foot control, improving the solo performance more than the dyad one -not requiring coordination between two agents- and thus reducing the difference between the solo and dyad performance. However if this result is present for any such constraints requiring coordination across the limbs represents an open question for further study.

### Subjective perception

Subjective perception in general is consistent with the quantitative analysis, although this perception may be biased by the reward of the score. Participants preferred the 3-coupled task and working in dyads. Both of these preferences were aligned with having a better score. Those results could be explained by the subjective preference for tasks that require lower cognitive load or by their preference for having higher reward from the score.

## Supplementary Information

Supplementary Information.

## References

[1] Lingard L, Reznick R, Espin S, Regehr G, DeVito I (2002). Team communications in the operating room: talk patterns, sites of tension, and implications for novices. Acad. Med..

[2] Abdi E, Burdet E, Bouri M, Bleuler H (2015). Control of a supernumerary robotic hand by foot: an experimental study in virtual reality. PLoS ONE.

[3] Di Pino G, Maravita A, Zollo L, Guglielmelli E, Di Lazzaro V (2014). Augmentation-related brain plasticity. Front. Syst. Neurosci..

[4] Wu, F.Y. & Asada, H.H. “hold-and-manipulate” with a single hand being assisted by wearable extra fingers. In *2015 IEEE International Conference on Robotics and Automation (ICRA)*, 6205–6212 (IEEE, 2015).

[5] Prattichizzo, D., Malvezzi, M., Hussain, I. & Salvietti, G. The sixth-finger: a modular extra-finger to enhance human hand capabilities. In *IEEE International Symposium on Robot and Human Interactive Communication*, 993–998 (2014).

[6] Parietti, F. & Asada, H. H. Supernumerary robotic limbs for aircraft fuselage assembly: body stabilization and guidance by bracing. In *IEEE International Conference on Robotics and Automation*, 1176–1183 (2014).

[7] Saraiji, M.Y., Sasaki, T., Kunze, K., Minamizawa, K. & Inami, M. Metaarms: body remapping using feet-controlled artificial arms. In *Proceedings of the 31st Annual ACM Symposium on User Interface Software and Technology*, 65–74 (2018).

[8] Parietti, F., Chan, K.C., Hunter, B. & Asada, H.H. Design and control of supernumerary robotic limbs for balance augmentation. In *IEEE International Conference on Robotics and Automation*, 5010–5017 (2015).

[9] Parietti, F. & Asada, H.H. Independent, voluntary control of extra robotic limbs. In *IEEE International Conference on Robotics and Automation*, 5954–5961 (2017).

[10] Hussain I, Spagnoletti G, Salvietti G, Prattichizzo D (2016). An EMG interface for the control of motion and compliance of a supernumerary robotic finger. Front. Neurorobot..

[11] Wu FY, Asada HH (2016). Implicit and intuitive grasp posture control for wearable robotic fingers: a data-driven method using partial least squares. IEEE Trans. Robot..

[12] Velloso, E., Alexander, J., Bulling, A. & Gellersen, H. Interactions under the desk: a characterisation of foot movements for input in a seated position. In *Human-Computer Interaction*, 384–401 (2015).

[13] Abdi E, Burdet E, Bouri M, Himidan S, Bleuler H (2016). In a demanding task, three-handed manipulation is preferred to two-handed manipulation. Sci. Rep..

[14] Dougherty, Z. & Winck, R.C. Evaluating the performance of foot control of a supernumerary robotic limb. In *ASME Dynamic Systems and Control Conference*, vol. 59162, V003T16A003 (2019).

[15] Huang Y, Eden J, Cao L, Burdet E, Phee SJ (2020). Tri-manipulation: an evaluation of human performance in 3-handed teleoperation. IEEE Trans. Med. Robotics Bionics.

[16] Winter, D. A. *Biomechanics and Motor Control of Human Movement* (Wiley, 2009).

[17] Balasubramanian S, Melendez-Calderon A, Burdet E (2011). A robust and sensitive metric for quantifying movement smoothness. IEEE Trans. Biomed. Eng..

[18] Wansoo, K., Lorenzini, M., Pietro, B., Yuqiang, W. & Ajoudani, A. Towards ergonomic control of collaborative effort in multi-human mobile-robot teams. In *IEEE/RSJ International Conference on Intelligent Robots and Systems* (2019).

[19] Peternel L, Fang C, Tsagarakis N, Ajoudani A (2019). A selective muscle fatigue management approach to ergonomic human-robot co-manipulation. Robotics Comput. Manuf..

